# Managerial strategies to make incentives meaningful and motivating

**DOI:** 10.1108/JHOM-06-2016-0122

**Published:** 2017-04-10

**Authors:** Sara Korlén, Anna Essén, Peter Lindgren, Isis Amer-Wahlin, Ulrica von Thiele Schwarz

**Affiliations:** 1 Medical Management Centre, LIME, Karolinska Institute, Stockholm, Sweden; 2 Center for Human Resource Management and Knowledge Work, Stockholm School of Economics, Stockholm, Sweden; 3 The Swedish Institute for Health Economics, Stockholm, Sweden

**Keywords:** Motivation, Professionalism, Health policy, Financial incentives, Patient-choice reform

## Abstract

**Purpose:**

Policy makers are applying market-inspired competition and financial incentives to drive efficiency in healthcare. However, a lack of knowledge exists about the process whereby incentives are filtered through organizations to influence staff motivation, and the key role of managers is often overlooked. The purpose of this paper is to explore the strategies managers use as intermediaries between financial incentives and the individual motivation of staff. The authors use empirical data from a local case in Swedish specialized care.

**Design/methodology/approach:**

The authors conducted an exploratory qualitative case study of a patient-choice reform, including financial incentives, in specialized orthopedics in Sweden. In total, 17 interviews were conducted with professionals in managerial positions, representing six healthcare providers. A hypo-deductive, thematic approach was used to analyze the data.

**Findings:**

The results show that managers applied alignment strategies to make the incentive model motivating for staff. The managers’ strategies are characterized by attempts to align external rewards with professional values based on their contextual and practical knowledge. Managers occasionally overruled the financial logic of the model to safeguard patient needs and expressed an interest in having a closer dialogue with policy makers about improvements.

**Originality/value:**

Externally imposed incentives do not automatically motivate healthcare staff. Managers in healthcare play key roles as intermediaries by aligning external rewards with professional values. Managers’ multiple perspectives on healthcare practices and professional culture can also be utilized to improve policy and as a source of knowledge in partnership with policy makers.

## Background

Healthcare systems worldwide face the challenges of increasing demands and limited resources. To drive efficiency, policy makers are introducing financial incentives and provider competition based on their presumed motivational effect. However, evaluation studies show inconclusive results (Fotaki *et al.*, 2008; Flodgren *et al.*, 2011), leaving both researchers and practitioners puzzled regarding how to improve policy. One part of this problem is the lack of conceptual models and empirical data that describe the process whereby the financial incentives are filtered through organizations to influence staff motivation. Although we can expect managers to play a key role in the translation and integration of incentives, their role has seldom been considered in previous research on incentives. The aim of the present study is to explore the strategies managers use as intermediaries between policy reform and staff motivation. We conducted an exploratory qualitative study of a local Swedish case that illustrates the general application of financial incentives and competition in healthcare markets. The study includes professionals in managing positions at healthcare providers involved in a patient choice reform in specialized orthopedics.

### Current trends in health policy

The policy application of market logics in healthcare is no longer predominantly a US phenomenon but is now widely applied in publically funded European healthcare systems as well (Roland and Rosen, 2011). This is certainly the case in Sweden, where this study is set (Anell, 2015; Harrison and Calltorp, 2000). Inspired by classic economic theory, competition between providers is encouraged by allowing patients to choose (Appleby and Dixon, 2004). Furthermore, financial-incentive models are developed to increase provider performance (Saltman, 2002). However, the empirical evidence is mixed and fragmented. Increased patient choice has been associated with increased provider performance in competitive markets (Cooper *et al.*, 2011), yet literature reviews provide limited support for stating that choice in itself improves efficiency or quality of care (Pollock *et al.*, 2011; Fotaki *et al.*, 2008). Similarly, review studies evaluating the impact of various financial-incentive models in healthcare conclude that the support for their effectiveness is limited (Flodgren *et al.*, 2011; Chaix-Couturier *et al.*, 2000). Incentives linked to specific provider behaviors, such as pay-for-performance-models, have shown to increase, e.g., productivity and cost efficiency (Eijkenaar *et al.*, 2013). However, concurrent reports of unintended consequences for patients (Chaix-Couturier *et al.*, 2000; Eijkenaar *et al.*, 2013) and healthcare professionals (Campbell *et al.*, 2007, 2008; Swarna Nantha, 2013; McDonald *et al.*, 2007) show negative side effects. Quality-based reimbursement (Conrad and Perry, 2009) and bundled reimbursement (Mechanic, 2011) have been proposed to avoid the pitfalls of narrowly defined performance targets. This aims to empower clinicians to drive quality improvement, but evaluations show both opportunities and challenges when it is implemented in practice (Mechanic, 2011). Overall, it is difficult to draw conclusions concerning the general effects of financial incentives due to methodological weaknesses of evaluation studies (Eijkenaar *et al.*, 2013; Flodgren *et al.*, 2011; Chaix-Couturier *et al.*, 2000).

In summary, whereas research exists regarding how the application of incentive models can be improved (Porter and Lee, 2013; Conrad and Perry, 2009), organizational characteristics, such as management structure, leadership and culture, are seldom considered (Frolich *et al.*, 2007). Indeed, there is a need for conceptual development and empirical data clarifying how externally imposed incentives are filtered through organizations and translated into motivation and behavioral changes at the individual level. In particular, a need exists for theory-based models that explore the hybrid role of managers in bridging the macro and micro levels of healthcare systems who align multiple, potentially conflicting sources of staff motivation. In the following section, we will draw on theories from the fields of psychology, sociology, behavioral economics and management to discuss the literature on individual motivation and the role of managers.

### Internal and external sources of motivation

Motivation is a multidimensional phenomenon defined as the energy and intention behind an action (Ryan and Deci, 2000). In contrast to classic economic theory underlying modern policy applications, motivation theories from different domains all take a broader approach to incentives. Both internal and external sources of motivation are acknowledged, and the interplay and potential conflicts between them are highlighted (Ryan and Deci, 2000; Swick, 2000; Ellingsen and Johannesson, 2007, 2008). Moreover, there is an agreement across fields that internal sources of motivation (that is, doing work for work’s sake) have a greater impact on behavior over time compared to external rewards (Deci *et al.*, 1999; Gneezy *et al.*, 2011; Ellingsen and Johannesson, 2007, 2008). There is also a convergence of motivational theories in that they often include basic human needs in terms of autonomy, expertise and pro-social behavior. In psychology, this is described in self-determination theory (Deci and Ryan, 2000). In sociology, theories of professionalism describe similar motivational forces that are highly applicable to healthcare. Professionals (an occupational group characterized by certain preferences) are described as being motivated by a high level of expertise (Freidson, 2001) and acting autonomously based on that expertise (Bøgh-Andersen and Holm-Pedersen, 2012). In addition, pro-social behaviors are also seen as a main component, as professionals are internally motivated to safeguard patient needs and social justice (Swick, 2000). Similar assumptions about motivational sources are reported in behavioral economics (Ellingsen and Johannesson, 2007).

### The interrelationship between multiple sources of motivation

Researchers from several fields have proposed that the coexistence of multiple sources of motivation can have a negative impact on overall motivation. The introduction of monetary rewards might harm or “crowd out” internal motivation and reduce overall performance (Ryan and Deci, 2000). Similarly, pro-social behavior has been shown to decrease in response to monetary rewards (Gneezy *et al.*, 2011; Benabou and Tirole, 2003). Some have argued that the risk of crowding-out effects is higher in public service such as healthcare due to the pro-social preference of staff (Frey *et al.*, 2013). There is empirical evidence to support the existence of a special kind of public service motivation (Bright, 2008) characterized by a stronger preference for internal reward, as compared to the private sector (Crewson, 1997; Georgellis *et al.*, 2011).

In contrast, there is also empirical evidence that external rewards can be highly motivating and “crowd in” overall motivation (Frey, 1994; Frey and Jegen, 2001). According to self-determination theory, the motivational effect of external rewards is dependent upon the extent to which it is internalized and consistent with the individual’s values (Deci and Ryan, 2000). Studies evaluating different forms of rewards show that the best motivational strategy involved combining personal monetary rewards with managerial feedback (Stajkovic and Luthans, 2003). These findings suggest that managerial behavior can play an important role in creating coherence between external rewards and internal values.

To summarize, in contrast to policy applications inspired by classic economic theory, research on motivation suggests that external incentives may reduce healthcare professionals’ motivation. However, contradictory findings indicate that external incentives may mobilize motivation when aligned with individual values. The presence or absence of alignment hence seems to be a key issue. In the next section, we discuss the role of managers as intermediaries potentially linking policy to the motivational preferences of their staff.

### The role of managers as intermediaries between external rewards and motivation

Management matters a great deal for organizational performance and staff motivation (Hales, 1999), including in healthcare settings (Lega *et al.*, 2013). Similarly, studies of line managers show that they play a key role in the implementation of new care processes (Dopson and Fitzgerald, 2006). According to traditional conceptualizations, managers’ primary role is to control staff through the planning, organization and coordination of tasks (Hales, 1999). More contemporary models of managerial behavior emphasize the reciprocal nature of management. In these models, managers are described as agents and facilitators between a network of stakeholders both inside and outside of the organization (Hales, 2002).

The number of managers employed to bridge the gap between top management and professionals has increased in healthcare (Kuhlmann and von Knorring, 2014). The rise of managerialism has conventionally been regarded as conflicting logic in professional organizations (Brommels, 2010), but research shows that hybrid managerial roles held by professionals is a common phenomenon (Kulhmann *et al.*, 2013). Hybrid managers enable professional and managerial logic to be intertwined by facilitating interaction between multiple stakeholders in organizations (Postma *et al.*, 2015). Such integration has been shown to improve both organizational performance and quality outcomes. Thus, the mere presence of management is insufficient, but management being informed by professional knowledge and values may have positive effects (Lega *et al.*, 2013). However, more knowledge is necessary on the specific strategies that managers use to balance various stakeholder perspectives in healthcare (Kuhlmann and von Knorring, 2014).

The central task of coordinating different stakeholder perspectives in healthcare has been described as articulation work (Strauss, 1988; Corbin and Strauss, 1993). Articulation work includes coordinating and fine tuning all tasks that emerge from the care process and comprises the communication and alignment of different stakeholder perspectives (Grant *et al.*, 2015). Thus, it is not a separate management process but rather an integral component of healthcare management based on professional expertise and culture (Postma *et al.*, 2015). Despite the centrality of articulation work in healthcare, its role in adapting to new policy is still largely unknown (Postma *et al.*, 2015).

To summarize, one central managerial behavior is to align the needs of different stakeholders by capturing perspectives ranging from the macro to the micro level. Building on this, we propose that the role of managers is central to policy implementation, and their strategies merit further exploration and analysis.

### Toward a tentative conceptual model

**Figure 1:**
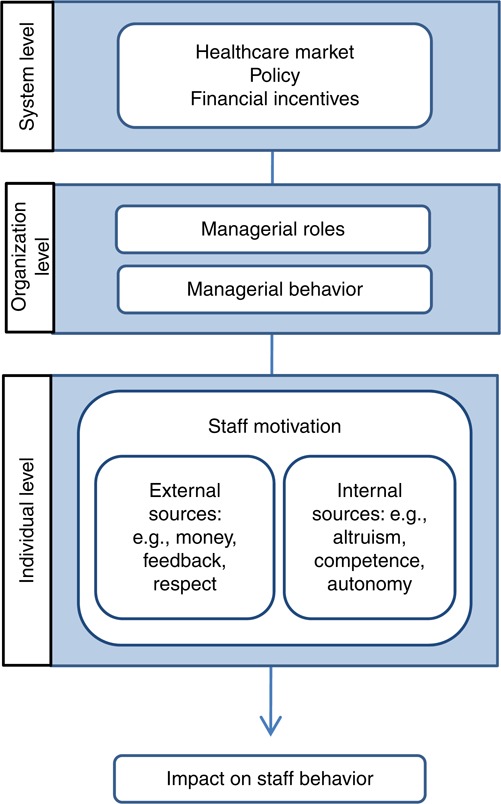
A tentative conceptual model illustrating the role of managers as intermediaries connecting external rewards at the policy level and sources of motivation at the staff level

The literature on individual motivation and management from the fields of psychology, sociology and behavioral economics is summarized in Figure 1. The conceptual model in Figure 1 suggests that managers can be understood as key intermediaries who use strategies to align the interests and perspectives of different stakeholders (e.g. policy makers, the organization and staff). It focuses on the role of managers and does not cover other contextual aspects that may mediate the relationship between external rewards and the individual motivation of staff.

## Methods

We conducted an explorative qualitative study of market-inspired patient-choice reform in specialized orthopedics based on interviews at six healthcare providers.

### Setting

The study targets a patient-choice reform in specialized care for hip and knee replacement that was introduced in 2009 and is still active. The County Council of Stockholm, a politically elected regional authority with responsibility for healthcare provision (Anell, 2015) for approximately 2.2 million people, introduced the reform. Acknowledging that governance commonly refers to a broad concept of the regulatory functions of a healthcare system (Kuhlmann and Burau, 2008), this reform includes a specific governance model built on specialization of care and provider competition. The reform includes a bundled reimbursement model that allows clinical freedom in designing the episode of care. The specific governance model of this reform will henceforth be referred to as “the model.” The model applies to both hospitals and specialized private providers with no productivity limits. The main reasons behind the reform were to increase providers’ competition through patient choice and lower waiting times for surgery. Evaluations of the effects have shown increased productivity and access to care (Vårdanalys, 2014).

The model covers a well-defined episode of care in hip and knee replacements for low-risk patients only. The selection of low-risk patients is performed using the American Society of Anesthesiologists (ASA) classification guidelines. Low-risk patients (ASA 1 & 2) may be treated by their chosen provider. However, high-risk patients (ASA 3 & 4) are excluded from the model and only handled by hospitals. Providers are reimbursed with a bundled payment to cover costs for the entire episode of care, including the final assessment for surgery, brief post-operative care and follow-up. Providers are financially responsible for all complications within two years after surgery, such as reoperations and infections. However, if infections occur, care must be provided at a hospital. If a specialized provider performed the initial surgery, they will be held accountable for the cost of care the hospital provided.

### Procedures

We invited all providers involved to participate in the study by contacting operation managers through e-mail and follow-up phone calls. In dialogue with operation managers at six volunteering providers, we used a purposive sampling approach to recruit respondents for interviews. In total, 17 interviews were held between June and November 2014, each lasting 45-60 minutes. In all, 16 interviews were conducted face-to-face and one over the phone. All were recorded with a digital audio recorder. All respondents were informed about the study both orally and in writing. They were told that participation was voluntary, and they all gave their written informed consent. All respondents were informed that the focus of the study was their personal experience and were encouraged to share their personal reflections. A semi-structured interview guide was developed to address three main themes: the respondents’ understanding of the model, managerial strategies to manage the implementation of the model and the respondents’ understanding of their staff’s motivation. The semi-structured interview guide included open-ended questions; examples of questions are “In what way do you adapt your leadership in relation to the model?” and “Is there anything you do to motivate your staff to engage in activities required to make the model work in practice?” Respondents were encouraged to freely expand their reasoning, as the guide did not include pre-defined follow-up questions. The interview guide was piloted in the first two interviews of the study. Based on the respondents’ feedback, the interview guide was adjusted by making questions more specific and shortening the list of pre-defined questions, leaving more room for the respondents’ own reflections. The first author conducted all interviews. The local ethical committee authorized the study (ref. no. omitted for review).

### Study population

We aimed to include different provider types in the study to obtain broad knowledge about how the same model works in different provider contexts. Two specialized providers and four hospitals volunteered to participate in the study. Two specialized providers declined to participate due to extensive internal reorganization. The participating specialized providers and one hospital are for-profit organizations. The county council owns and runs the remaining three hospitals. All participating hospitals also handled hip and knee replacements for high-risk patients under a separate provider contract. In dialogue with the operation managers, we recruited healthcare professionals in managerial positions handling the episode of care covered by the model. We aimed for a broad representation of professionals and reached out to both nurses and orthopedic surgeons in managerial positions. The operations managers were also asked to participate. In total, 18 people were asked to participate, and of these, 17 agreed to participate. The respondents’ respective roles were operations manager (six), clinical manager (five), manager of post-operative care (three), operations coordinator (one), quality manager (one) and research manager (one). All respondents had substantial clinical experience as nurses (five) and orthopedic surgeons (12), and all were clinically active.

### Data analysis

All interviews were transcribed verbatim and analyzed in NVivo. Data familiarization occurred concurrent with interviewing. After completing the majority of the interviews (15), data saturation was reached. The final two respondents were recruited to control for additional themes, but no new themes were discovered. After the in-depth immersion of data, we applied a two-step data-analysis process using a hypo-deductive approach (Fereday and Muir-Cochrane, 2008). First, the first author made a deductive abstraction of a focused data set. Guided by general definitions of managerial behavior (Hales, 1999), data of interest were identified throughout the data corpus. To minimize the risk of bias, the last author validated the abstracted data through independent identification of relevant data in three sampled interviews. In total, 85 percent of the identified data overlapped perfectly; all remaining inconsistencies were discussed until a consensus was reached. The second step of the analysis focused on the data set addressing managerial behavior using a thematic analysis with an inductive approach (Braun and Clarke, 2006). The remaining data corpus served as background information and provided a deeper understanding of managerial behavior. First initial codes such as “avoid communicating cost” and “talk about patient value” were generated. Thereafter, the codes were clustered to identify initial themes (e.g. “adapting,” “sense making”). Initial themes were reviewed and revised iteratively, eventually resulting in defining and naming final themes, i.e., the strategies of managers. The first author compiled the inductive thematic analysis, repeatedly discussing it with colleagues. The second and last authors validated the final themes through independent reading of three interview transcripts. The accuracy and completeness of the final themes in relation to the data were examined, and all comments were discussed until a consensus was reached.

## Findings

**Figure 2:**
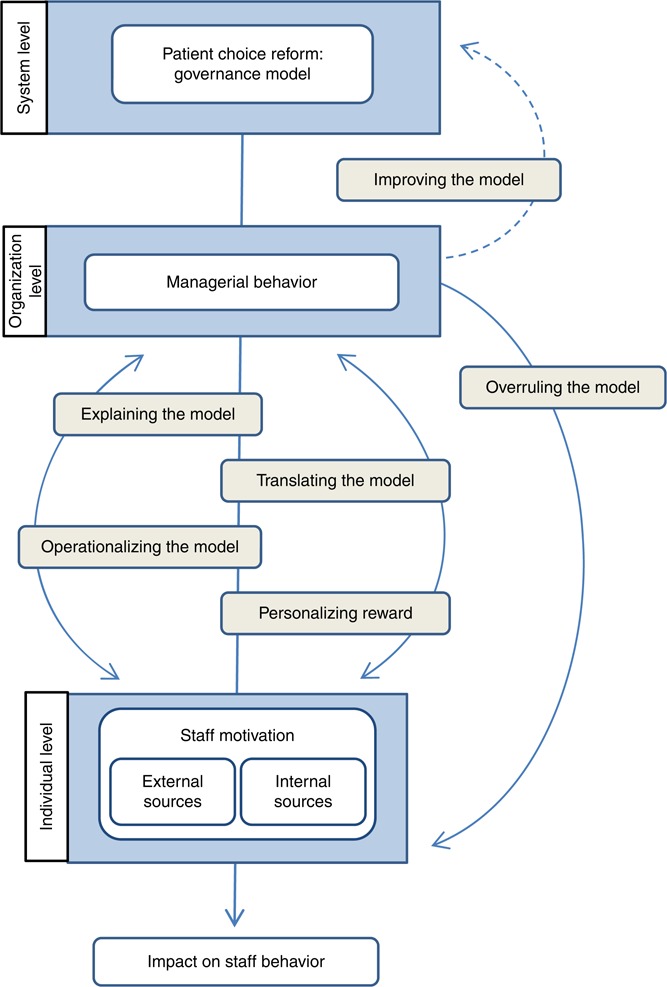
Managerial alignment strategies

The analysis confirmed that the managers considered themselves intermediaries between the governance model and their staff. The respondents described four major alignment strategies they applied in their roles as managers. The aim of these strategies was to reconcile the requirements of the model and their staff preferences. One additional strategy, which involved overruling the model, was used more rarely in situations in which staff motivation was at risk of being reduced. One proposed but unrealized strategy was to improve the system through a dialogue with policy makers. The six strategies are described below, preceded by a description of the contextual knowledge possessed by the managers – knowledge that was a key prerequisite for their efforts to contribute to alignment (see Figure 2).

### Contextual knowledge

The respondents’ managerial strategies were based on knowledge obtained in their hybrid roles as both managers and professionals. This knowledge was a key precondition for their ability to formulate successful alignment strategies in this specific organizational context. The managers expressed a broad, experienced-based knowledge of the model’s consequences and had a clear picture of what kind of changes the model required to maintain a financially viable organization. In general, the model put pressure on providers to work more efficiently: the reimbursement levels were lower, and they were held financially accountable for any complications to provide an incentive for high-quality care. The managers also had an understanding of how the model and the changes it required affected the organization of care, the working conditions of the staff and the experiences and outcomes of the patients at their clinic. This in-depth understanding of the practical implications of the model guided managers in identifying appropriate opportunities for change and helped them formulate reasonable and realistic ambitions.

When devising strategies to achieve these ambitions, the managers’ understanding about what was needed to engage and motivate staff was key. Expressions such as “we” and “us” were used repeatedly when describing the motivational preferences of their team, indicating that staff and managers had largely the same preferences: concern for patient needs and ambition to provide high-quality care and the sensible use of resources. Professional pride in one’s expertise was also perceived as being central to staff, as was a sustainable work environment.

### Managerial alignment strategies

#### Explaining the logic of the model

The respondents tried to increase staff members’ awareness and understanding of the model by explaining its background and implications and framing it from a provider perspective and in relation to the healthcare system as a whole. They attempted to provide a rationale for the changes needed by explaining the reason for the model from a policy perspective. The respondents also tried to increase understanding of the model by framing it in relation to the role and mission of their own organization, contrasting it to other providers in the healthcare system. This was perceived as being more complicated for hospital providers, compared to private providers, due to the multiple provider contracts in operation at hospitals. They attempted to explain the limitations and opportunities the regulations implied, e.g., the selection criteria and reimbursement logics that the regulations entailed. The respondents described the need to continuously repeat and communicate the regulations to maintain awareness among staff:

It became sort of, okay so what’s the difference? It was not obvious, not at all. And then we had to go through the patient-choice model and see what was included, what was expected and what kinds of visits? Simply a learning process […]. What’s still problematic is that the physicians haven’t grasped this […]. They don’t know the conditions and can promise patients things that aren’t included

(Interview, Clinical manager, Hospital).

The respondents experienced challenges associated with explaining the model to their staff. The regulations were difficult to apprehend and communicate in an effective and understandable manner. They also expressed the challenges in communicating about a model that in itself evoke mild or no interest in sharp contrast to staff members’ engagement in care provision:

I think economics in health care is really hard to comprehend […]. And then, you might not be as interested in it. If you were, you’d have chosen to become an economist […]. In contrast, you’ve chosen to become a doctor because you’re interested in helping and caring for others and such things

(Interview, Clinical manager, Hospital).

#### Translating the model

To engage staff, the respondents applied a strategy of translating the economic logic of the model into goals and targets perceived to be in line with staffs’ motivational preference. This included a focus on patient value and outcomes. Thus, for example, initiatives to optimize the care flow were communicated as improvement initiatives:

I think you get engaged by that, if you present arguments about how value is created for patients. And how you can see this in your daily work; if you change something, then the patient can get out of bed one day earlier and recover, and can wear their own clothes two days after surgery, and look like a healthy person rather than one suffering from illness, that’s worth a lot. Things that are concrete, that you’re doing good and getting results. If you present such arguments and work to achieve such goals, then in my experience it’s easy to get things through. It’s harder if you give arguments like we have to cut down on resources and make restrictions

(Interview, Operations manager, Specialized provider).

Although the managers felt that patient-centeredness and high-quality care were the main motivational focus among the staff, staff members were not indifferent to resource use and costs. Particularly, the sensible and fair use of public resources was described as important. Talking about making a profit by dealing more efficiently with patients could, on the other hand, easily be perceived as provocative and a violation of professional ethics, which would increase resistance to change. The economic figures were also perceived as too loosely linked to staff control and therefore not functional as a motivational tool. The respondent often avoided using explicit economic figures in their communications with staff:

I have not said that now we have to do this because we get 10,000 SEK less per patient. I would never ever communicate in that way […]. That would never work as a carrot. If you want people to grow and contribute to change, then you’ll have to do it in a way that allows you to really maintain quality

(Interview, Clinical manager, Hospital).

When they discussed economic conditions, they felt it necessary to concurrently assure the staff that patients and quality come first. This was also of central importance to the managers themselves:

I think the challenge has been, on the one hand, to make staff and physicians aware of costs and to understand that we control this. But also, to clearly communicate that we put patient safety and quality first

(Interview, Operations manager, Specialized provider).

#### Operationalizing the model

The respondents described a strategy of operationalizing the logic of the model by breaking it down into well-defined, concrete and feasible work tasks. This was particularly important in creating opportunities for feedback, which the respondents experienced as an essential motivational tool that they used frequently to follow-up operationalized tasks. The respondents emphasized the importance of monitoring daily work, as this enabled them to give specific and credible feedback. Feedback was described as having a twofold positive effect on motivation: providing information about task and goal achievement, which was rewarding in itself given staff members’ desire to master their work and meet patient needs, and as an opportunity to show managerial support and praise.

The managers described using different sorts of data to provide feedback on operationalized tasks. Measures of patient outcomes and patient satisfaction were thought to have the strongest motivational impact on staff, whereas process measures were valued because they were easily linked to their contribution and performance. Information was captured in the daily work and dialogue with patients but also through regular follow-ups on outcome measures from the national quality registries. The respondents reported that professional pride associated with being a high-quality provider was motivating to staff and that benchmarking with other providers was important to inspiring future improvement work:

What would you say your team and staff are interested in when it comes to feedback and measures?

How the patients are doing and their experience. Some general sense of how much value we have provided for them

(Interview, Operations manager, Hospital).

#### Personalizing rewards

Across provider types, the respondents described a strategy of personalizing rewards to make them relevant at the individual level. They believed that rewards had to be suited to personal preferences and gave examples of how financial incentives could be rewarding for some individuals, whereas opportunities for research and competence development were more motivating for others.

There was no direct link between incentive logic at the provider level and the individual payment of staff, but one private provider attempted to personalize economic rewards by introducing a team-based quality bonus for all staff members (for reducing complications costs). The respondents, across provider types, emphasized that non-monetary personal rewards could also be highly motivating – if the individual valued them. In the experience of the managers at public providers, assigning time off for staff to engage in research and competence development had proven to be an appreciated reward. The respondents at private providers who had limited involvement in research expressed the importance of giving private-sector staff opportunities for competence and skill development to support their long-term career development:

So, there is a certain group of physicians who are more interested than others in making money and less interested in professional competence development. Sometimes you see both, but there are differences for sure. Here, we have traditionally applied a fixed monthly pay, and in my experience, my colleagues are more interested in getting a reputation as skilled physicians and gaining the respect of others as well as in caring for the patient’s wellbeing. To have that as a driving force

(Interview, Operations manager, Specialized provider).

#### Overruling the model

The respondents reported occasionally finding themselves forced to overrule the model by diverging from its economic logic and performing actions that did not maximize their provider organization’s financial gain. This occurred in situations where a patient needed actions that were not covered by the reimbursement. To avoid the risk of harming patients and thereby also weakening staff motivation, the managers decided to act at the provider’s own expense. Examples of overruling the system were described across provider types. According to the respondents, this was most commonly caused by flaws in the inclusion assessment procedure (ASA), which does not consider psychiatric status or age as risk factors. This procedure results in patients being classified as low risk, despite requiring more extensive post-surgery care. The provider organization then financed longer rehabilitation.

The respondents gave several reasons for overruling the system. They referred to their own ethical convictions as a health professional that hindered them from leaving patients to suffer. They also used this strategy to protect staff well-being and motivation because they were aware of staff members’ professional values and concern for patients:

Yes, I believe this is important. I think it’s important since we work so close to people all the time, and if we were to let compliance with the system become more important than the patients, then I think that you wear your staff down, then you lose your energy. We can’t stand that […] I think it’s a self-preservation strategy for the health care community, that we stay united

(Interview, Clinical manager, Hospital).

Some respondents expressed concern about becoming trapped in a conflict between their professional and managerial roles by representing a rigid model that they did not fully support. They described the lack of opportunities to change the model as a source of frustration, potentially putting their own work motivation at risk. Other respondents described feeling partly disillusioned, as they had stopped believing that dialogue and change were possible.

#### Improving the model at the policy level

The respondents described wanting but not having a strategy to improve the model design through dialogue with policy makers, the aim being to better align the model with patient needs and professional motivation. They wanted to engage in dialogue with policy makers to share their insights into the practical implications of the model for staff and patients and discuss potential improvements. They called for a formalized forum for feedback between policy makers and professional representatives from local providers instead of the current system of only using a national expert reference group. The respondents described occasional contact between providers and regional decision makers, but these conversations were focused on the management of specific patients rather than on the overall model design:

Yes, there’s an ongoing dialogue with the county council administration and continuous revision of the rules and regulations […] Unfortunately I’m not involved; instead there’s a bunch of national experts in orthopedics who are represented there and that I find unreasonable. And I have expressed my opinion on that; why aren’t those of us working with this model part of that? We know how it works, what doesn’t work and what could be improved

(Interview, Operations manager, Specialized provider).

The respondents also thought a closer dialogue with policy makers could enable the proactive co-creation of better governance models in the future. They said that components that appealed to professionals could be used more actively, e.g., incentives closely linked to quality and earmarked reimbursement for education and research activities. The respondents also suggested that increasing the model’s flexibility based on trust in professionals would improve the system for both professionals and patients.

## Discussion

The present study shows that the managers indeed play an intermediary role in connecting the policy and staff levels of healthcare systems and that they use several alignment strategies to make the governance model studied more motivating to staff. The strategies they employ are based on their knowledge of the practical consequences of the governance model at the provider level, but the strategies are also formulated in relation to the perceived motivational preferences of the staff.

The literature on motivation suggests that although external rewards, such as financial incentives, may crowd out staff motivation (Benabou and Tirole, 2003; Gneezy *et al.*, 2011), they may also “crowd in” motivation if they are aligned with the underlying values of the individual (Frey and Jegen, 2001). The results presented here confirm the role of managers in linking and aligning internal and external sources of motivation (Deci and Ryan, 2000), as outlined in the tentative conceptual model we provide (Figure 1). The empirical data adds to this by showing how managers use different alignment strategies to make this happen (see Figure 2). The strategy of personalizing rewards refers to managers’ efforts to complement the model with additional rewards adjusted to the motivational preferences of each individual. This strategy highlights an awareness of the variability of individual preferences and managers’ intuitive understanding of the important role of intrinsic motivators, such as enabling competence development, to maintain a satisfactory level of motivation over time. The strategy of explaining the model refers to the rhetorical work managers perform to make sense of the model and create awareness among staff concerning its practical implications. Beyond that, the managers translate the model into motives and arguments related to patients, thus supporting staff members’ assumed internal, pro-social motivation. The strategy of operationalizing the model and using feedback may also be regarded as a way of supporting the internalization of staff. By giving feedback on how the staff contributes to value creation and overall goal of the organization, the managers align external rewards to staff members’ internal motivation of competence and expertise. Together, the strategies are used to align the model with professional values, which relates closely to the internalization process presented in self-determination theory. It is also in agreement with the literature on professionalism and public service motivation. The strategy of overruling the model to prevent economic reward from becoming too dominant and weakening staff members’ internal motivation is particularly noteworthy in this regard – managers are willing to take financial risks to minimize the risk of the crowding-out effect.

In summary, our findings demonstrate that externally imposed financial rewards are not motivating on their own and that managers play a vital role in articulating the ways in which incentives align the motivation of healthcare staff. The empirical data show how managers transform the inbuilt orientation toward cost-effectiveness of financial reforms into new meanings that appeal to healthcare professionals.

By explaining the model to staff and calling for a dialogue with policy makers, the managers in this study bridge the gap between the micro and macro levels of healthcare. This extends previous research on articulation work in healthcare provision (Corbin and Strauss, 1993) by explicating strategies used in the policy articulation process. Whereas many of the managerial strategies identified here involve articulation work focused on staff, the improvement strategy goes in the opposite direction, targeting policy makers to change the model to indirectly affect staff motivation. The different directions of the strategies raise questions concerning managers’ choice of strategy and how different stakeholder perspectives are prioritized and judged – questions that merit further exploration. In particular, the alignment strategies employed by non-medically educated managers would be relevant to study.

### Implications for practice

The present study has practical implications for both healthcare provision and health policy. First, healthcare providers may in different ways support managers in applying alignment strategies. In addition to time and knowledge, administrative systems with access to high-quality data on patient outcomes and resource use could increase their opportunities to give meaningful and thus motivating feedback. Providers could also maintain a dialogue with decision makers at a policy level, thus increasing opportunities for the improvement of policies. The present results further support the benefits of hybrid roles in healthcare management (Kuhlmann and von Knorring, 2014), which could guide provider organizations in their recruitment and development of management.

Second, our findings suggest that the way health policies and governance models are designed may influence managers’ abilities to form alignment strategies. Policies that support high quality of care will be easier for managers to align with staff motivation and more likely affect staff behavior and provider performance. Policy makers could also inform about new policies and governance models in a timely fashion and provide argument for the reform referring to patient and staff benefits, not only efficiency.

Our study also provides perspectives on how the process of designing policies and governance models can be improved. Healthcare is typically described as a complex adaptive system involving interaction between multiple agents and resulting in low predictability. Deviations from standardized procedures are everyday occurrences, and flexible control systems have been recommended (Sturmberg *et al.*, 2012). It may be naïve to assume that anyone, policy makers included, can foresee all potential consequences and design optimal models. As Casalino (Casalino, 1999) noted, the inbuilt incompleteness of measures to define and control healthcare processes makes unintended consequences inevitable. The present results suggest that the introduction of new policies and governance models should be regarded as a continuous process of change rather than episodic, which requires constant adaptation to a complex environment (Weick and Quinn, 1999). In complex settings, the co-production of services involving several stakeholders sharing a common goal has been shown to be essential. This approach is increasingly applied in healthcare services, e.g., in patient-centeredness and self-management (Batalden *et al.*, 2015). We propose that policy makers and professional managers form partnerships to co-produce policies, aligning stakeholder perspectives and enabling continuous improvement over time. To summarize, this requires a shift from efforts to design the best model to efforts to design the best process to improve the model through co-production.

### Methodological considerations

The present study has limitations that require consideration. The study was conducted in specialized orthopedics, in which managers handled this specific governance model. The managerial strategies identified in this study needs to be further explored in in other healthcare settings and systems, including more complex diagnoses, different market compositions, provider structures and different incentive models. Furthermore, the body of literature guiding the focus of this study mainly takes an individual perspective on motivation and management. Particularly, system dynamics at the micro, meso or macro level needs to be investigated in future studies.

Nevertheless, our findings show similarities across groups of respondents from different provider types (private and public) who have different professional backgrounds (physicians and nurses) and roles (operations manager, clinical manager, etc.). This supports the relevance of the identified managerial strategies. To uncover the generalizability of strategies across e.g., provider types and professional groups is beyond the scope of this study but merits further exploration. In light of the expressed need for comprehensive theoretical models in health policy research (Frolich *et al.*, 2007), we hope the tentative conceptual model presented here will be found helpful in this regard.

Regarding the validity of our results, the fact that we rely on data that only managers have provided should be considered. We have not interviewed staff or policy makers, and thus, our results reflect the views of managers rather than an objective “truth.” We have not made observations to assess managerial behaviors and thus have a limited knowledge of when and to what extent the described strategies are used. More research is needed to include complementary perspectives and additional data sources illustrating the relationships between managerial strategies and their impact on motivation.

Two operations managers from specialized providers declined to participate, and our sample includes a slightly lower proportion of respondents from specialized providers. There is a risk that our findings are colored by a selection bias in our sample of volunteering respondents. We have not been able to control to what extent our respondents have had formal training or varying skills in implementing policy reforms of this kind. There is also a risk that our respondents are more favorable or critical to policy compared to managers in general, which could affect their strategies. In summary, our empirical result highlights the need to further address the interaction between stakeholders at the policy and provider levels.

## Conclusion

The present study aimed to explore managers’ strategies as intermediaries between policy reform and staff motivation using empirical data from a local case study. The main conclusion is that managers have a central role to play in making policy motivational in practice and potentially buffering negative consequences at the staff level. Their knowledge and awareness of multiple perspectives on healthcare provision and professional culture could also be used in the co-production of future health policies. Managerial and professional logic should be integrated with the demands and logic of health policy. Such a partnership could realize the creation of policies that support high-quality care and efficient resource use, which has been found to motivate healthcare professionals.
